# Being “in” or “out” of the game: subjective and acoustic reactions to exclusion and popularity in social anxiety

**DOI:** 10.3389/fnhum.2014.00147

**Published:** 2014-03-18

**Authors:** Eva Gilboa-Schechtman, Lior Galili, Yair Sahar, Ofer Amir

**Affiliations:** ^1^Department of Psychology and the Gonda Multidisciplinary Brain Research Center, Bar-Ilan UniversityRamat Gan, Israel; ^2^Department of Communication Disorders, Sackler Faculty of Medicine, Tel Aviv UniversityTel Aviv, Israel

**Keywords:** social phobia, rejection, acceptance, self-esteem, dominance, social rank, acoustic analysis, voice

## Abstract

Social Anxiety (SA) has been shown to be associated with compensatory deficits in pro-social behavior following exclusion and with failure to capitalize on social success. We assessed the subjective and expressive responses of high (*n* = 48) and low (*n* = 56) socially anxious individuals to exclusion, acceptance, and popularity induced by a participation in an online ball-tossing game. Before the manipulation, participants read aloud neutral and command utterances. Following the manipulation, participants rated their mood and cognitions and re-read the utterances. Acoustic properties (fundamental frequency–mF0, vocal intensity) of these utterances were analyzed. We found greater differences in self-esteem between high and low socially anxious individuals following the exclusion condition, as compared to the acceptance condition. Among low socially anxious individuals, exclusion promoted increased vocal confidence, as indicated by decreased mF0 and increased vocal intensity in uttering commands; High socially anxious individuals exhibited an opposite reaction, responding to exclusion by decreased vocal confidence. Following popularity, high SA was associated with decreased enhancement in mood and self-esteem in women but not in men. Consistent with evolutionary and interpersonal accounts of SA, we highlight the importance of examining the effects of SA and gender on events indicating unambiguous and unanimous social acceptance. Examining reactivity to changes in belongingness may have important implications for understanding the core mechanisms of SA.

## Introduction

Social Anxiety disorder (SAD, or social phobia) is a condition involving marked anxiety about social or performance situations in which there is a fear of embarrassing oneself under scrutiny by others (DSM-IV, American Psychiatric Association, 2000). SAD often has its onset in childhood and tends to precede most other disorders with which it is co-morbid, most notably depression (Bittner et al., 2004). SAD is associated with severe psychological, interpersonal, and professional consequences (e.g., Ruscio et al., 2008). Given these anxieties and avoidances, it is not surprising that socially anxious individuals report high levels of negative affect, and functional impairment in several life areas (Aderka et al., 2012). Unsurprisingly, SAD is also associated with lower wellbeing (Sherbourne et al., 2010) and lower levels of positive affect (e.g., Kashdan, 2007). These findings have frequently been related to the impairment in interpersonal connectedness common in SAD (e.g., Gilboa-Schechtman et al., 2013a,b).

Most theoretical models of social anxiety (SA) consider heightened sensitivity, enhanced responsivity, and impaired affective regulation in the face of social threat to be at the epicenter of this condition (e.g., Clark and Wells, 1995; Rapee and Heimberg, 1997; Gilbert and Trower, 2001; Hofmann et al., 2004). During human evolutionary history, loss of belongingness was associated with threat to survival (Wesselmann et al., 2012a,b). Accordingly, the human tendency to belong and affiliate is frequently defined as one of the most essential and fundamental needs (e.g., Baumeister and Leary, 1995). Given the centrality of belongingness, basic psychological systems are postulated to monitor for changes in social inclusion and exclusion. Sensitivity to changes in belongingness is frequently explained in evolutionary terms: being a member of a group improves survival chances due to the protection and resources offered by the group (Lancaster, 1986). Relatedly, positive affect experienced in response to social acceptance is likely to strengthen one's psychological resilience (Fredrickson et al., 2003), to promote physical health (e.g., Davidson et al., 2010; Boehm and Kubzansky, 2012), and to increase longevity (Xu and Roberts, 2010).

Consistent with these theoretical postulations, social exclusion has been found to provoke significant changes across multiple psychobiosocial domains. It has been found to engender subjective experience of distress (Van Beest and Williams, 2006), behavioral dysregulation (Oaten et al., 2008), changes in cognitive efficiency (Hess and Pickett, 2010), changes in attentional focus (Dewall et al., 2009), enhanced blood pressure (Stroud et al., 2000), cortisol reactivity (Blackhart et al., 2007) and enhanced activation in brain regions that process and regulate the unpleasantness of physical pain (Eisenberger et al., 2003). The salubrious effects of social acceptance are also robust. Social acceptance is associated with changes in mood, self-esteem, behavior and physiology (e.g., Leary et al., 2001; Mendes et al., 2008; DeWall et al., 2010). Yet, given the centrality of the belongingness system, its implications to psychopathology in general, and to SA in particular, have not been thoroughly explored. This is the main theme of the present research.

SA is postulated to function as a warning system that alerts people to potential threats to their belongingness status (Leary and Kowalski, 1995; DeWall et al., 2011). Indeed, it has been found that socially anxious individuals are characterized by a high sensitivity to exclusion (Zadro et al., 2006). Specifically, Zadro and her colleagues found SA to be associated with more prolonged recovery following an exclusion manipulation. Using a similar exclusion paradigm, Oaten and colleagues found that individuals with high SA differ from individuals with low SA in their ability to self-regulate following exclusion (Oaten et al., 2008). Further, research in temperamentally shy children found more intense emotional upheaval and poorer vagal regulation in response to peer rejection (Gazelle and Druhen, 2009). Moreover, in a recent study with children, Reijntjes and colleagues found that SA was associated with greater changes in state self-esteem following peer disapproval (Reijntjes et al., 2011).

It appears that SA affects not only the quantitative, but also qualitative nature of coping with exclusion. While among low-socially anxious individuals exclusion promoted renewed interest in connecting with sources of positive social interaction, high-socially anxious individuals failed to react to rejection in a prosocial manner and exhibited evidence of decreased social interaction effectiveness (Mallott et al., 2009). Specifically, Mallott and colleagues examined nonverbal characteristics of self-presentation of individuals high and low in SA following interpersonal rejection. They found that, observers' subjective ratings of vocal and eye-gaze performance was inversely related to SA. In the present study we sought to extend the investigation of the effects of changes in social belongingness, to include objective measures of vocal production. Acoustic analysis of speech is emerging as an indirect, noninvasive, and sensitive measure of emotional state (Elfenbein and Ambady, 2002; Juslin and Laukka, 2004) and interpersonal strategies (Bugental et al., 2009), in research as well as in clinical settings (Diamond et al., 2010).

Vocal parameters have been examined in an attempt to capture the emotional “tone” of the voice—that is the aspect of speech that is not conveyed through the meaning of verbal utterance. These nonverbal features of a spoken message (Tusing and Dillard, 2000) have been shown to play an important role in conveying emotions (Laukka and Elfenbein, 2011) and in conducting power negotiation (Scherer, 1986; Scherer et al., 2003). Vocal parameters are less controllable than are other types of nonverbal behaviors (Zuckerman et al., 1981) and therefore may serve as “honest signals” of the speaker's current emotional state (Bugental et al., 2009). The vocal parameters that have been most frequently used in past research are fundamental frequency (mF0) and vocal intensity.

There is a robust line of research linking certain parameters of vocalization to social rank. Consistent with Ohala's (1982) evolutionary model, lower mF0 has been associated with enhanced dominance (e.g., Ohala, 1984; Puts et al., 2006, 2007; Jones et al., 2010). Vocal intensity is positively associated with dominance rating in the production of spontaneous speech (Tusing and Dillard, 2000). Moreover, these parameters were also shown to differentiate between vocal profiles of different intents (Galili et al., 2013). Specifically, as compared to neutral utterances, command utterances were characterized by increased mF0 and higher vocal intensity. Acoustic analysis has the potential to offer a subtle understanding of the ways in which individuals negotiate interpersonal interactions. Yet, acoustic analysis has, until recently, been under-utilized. We believe it offers a way to understand corrective actions people take following exclusion.

Measures of acoustic production show promise as indirect measures of SA (e.g., Laukka et al., 2008; Weeks et al., 2011, 2012; Galili et al., 2013). Specifically, analyzing the vocal properties of planned speech, we found that SA was associated with higher mF0, and with decreased vocal intensity in men (Galili et al., 2013). Using spontaneous speech, Weeks and colleagues similarly found that clinical SA was associated with increased F0, and that this pattern was more pronounced in men than in women (Weeks et al., 2012). In addition, Laukka and colleagues found that among clinically socially anxious individual mF0 was decreased among treatment responders (Laukka et al., 2008). In view of these findings, the primary aim of the present study was to extend the research on reactivity to social exclusion in SA by including acoustic indices of interpersonally-directed utterances.

The second aim of this study was to examine the reactions of socially anxious individuals to events connoting social acceptance. While those events are commonly experienced as positive by nonsocially anxious individuals, this is not necessarily the case for socially anxious persons (e.g., Weeks and Howell, 2012). Several perspectives (e.g., Alden and Taylor, 2004; Weeks and Howell, 2012; Gilboa-Schechtman et al., 2013a,b) converge in suggesting that socially anxious individuals may exhibit biased processing of positive social attention. There is growing evidence indicating that socially anxious people were less successful at capitalizing on positive social experiences than are individuals without SA, even after controlling for depression (e.g., Gilboa-Schechtman et al., 2000; Kashdan et al., 2011; see also Gilboa-Schechtman et al., 2013a,b, for review). Exploring the nature of socially anxious individuals' reactions to events indicating social acceptance is likely to contribute to the greater understanding of core processes in SA.

The experimental research on the effects of positive social attention in SA has been limited. In a pioneering study, Alden and colleagues found that, upon receiving positive feedback following a social interaction, individuals with high levels of SA expected to experience greater levels of anxiety regarding a future social interaction (Alden et al., 2004). In addition, following the receipt of positive feedback, people with high levels of SA predicted that their partner would expect more from them in the next interaction, and that they would fall short of those expectations (Alden and Wallace, 1995; Wallace and Alden, 1997). Finally, Alden and colleagues found that the tendency to interpret positive social events as indicative of negative future outcomes partially mediated the relationship between SA and decreased positive affect (Alden et al., 2008). Importantly, in all of these studies success in a given interaction was found to bear on future interaction. But what if the “beam of social attention” was not specifically related to future occurrences? Does social visibility exert a “warm glow” for socially anxious and nonsocially anxious people alike? Addressing this question was the second aim of our study.

In the present study we assessed the subjective and expressive responses of individuals high and low in SA to exclusion, acceptance, and popularity induced by a participation in an online ball-tossing paradigm—Cyberball. Cyberball is one of the most commonly used procedures in investigating the effects of social exclusion (Williams, 1997, 2001, 2009). Previous Cyberball studies included two conditions: exclusion vs. acceptance (i.e., receiving a “fair share” of the throws). In the present study we introduced a third condition—popularity—in which participants received all the possible throws from the other two players.

Before beginning the Cyberball task, participants read aloud neutral, command and request utterances. Upon completing the Cyberball task, participants rated their mood and cognitions and re-read aloud the utterances. Subjective, cognitive, and acoustic measures (mF0, vocal intensity) were analyzed.

Four hypotheses were examined. First, consistent with the enhanced exclusion-reactivity accounts, we postulated that as compared to low SA individuals, individuals high in SA would report lower mood and self-esteem following exclusion as compared to acceptance (enhanced exclusion reactivity hypothesis). Second, consistent with the impaired positivity account (Gilboa-Schechtman et al., 2013a,b), we postulated that, as compared to individuals low in SA, individuals high in SA would report lower mood and self-esteem following popularity as compared to the acceptance condition (impaired positivity hypothesis). Third, with respect to the acoustic parameters, consistent with the compensatory deficits view of SA, we postulated that exclusion (as compared to acceptance) would lead to more insecure (and less dominant) behaviors in individuals with low levels of SA, while individuals high in SA would not exhibit this pattern. Specifically, we expected to observe a greater increase in mF0 and a greater decrease in vocal intensity for command vs. neutral sentences in individuals high in SA, as compared to individuals low in SA (vocal insecurity following exclusion hypothesis). Fourth, we also expected that following popularity, individuals high in SA would exhibit a lesser increase in a pattern of confident vocal behavior as compared to individuals low in SA. Specifically, we expected a smaller decrease in mF0 and a smaller increase in vocal intensity for command vs. neutral utterances in individuals high in SA, as compared to individuals low in SA (vocal confidence following popularity hypothesis).

## Methods

### Participants

Hundred and four university students (58 women) took part in the study in exchange for 30 NIS (equivalent to 8 US$) or academic credit. Participants were recruited through the Bar-Ilan University Psychology Department Subject Pool, as well as from advertisements in billboards on campus and electronic forums. Before arrival to the laboratory, participants received several self-report questionnaires, including questionnaires assessing SA. Participants who scored below the accepted cut-off for clinical range or above the cut-off for diagnosis for SAD (Baer and Blais, 2010) on a self-assessment measure of SA (Fresco et al., 2001) were invited to participate in the study.

### Procedure

Participants were invited to take part in a study investigating individual differences in “visual perception and vocal production.” Upon arrival to the laboratory and signing a consent form, participant met a confederate who was introduced as a fellow participant. Participants were introduced to the research purpose and procedure, and were photographed using a web camera for future use in the Cyberball task. Next, participants engaged in a first (pre-manipulation) vocal recording session.

Participants were then told that they will play an internet game “Cyberball” (see Williams et al., 2000) with two other students, one of whom they already met in the waiting room, and the other is waiting in an adjacent lab. Next, the experimenter made a staged phone call to the neighboring laboratory, informing that the participants (the confederate and the actual participant) are ready to start. Participants were randomly assigned to one of three conditions in the ball-tossing game: Exclusion, Acceptance, and Popularity. In all conditions, the game lasted approximately 5 min.

Upon completion of the game, participants filled out the Basic Needs Threat Questionnaire (Zadro et al., 2004). Next, they performed the second (post-experimental) vocal recording session. Then, they took part in a brief (3 min) cognitive task not reported in the present study. All participants then completed several self-report questionnaires. Lastly, they were de-briefed by the experimenter about the real purpose of the experiment and its procedure. During the debriefing participants were interviewed about the believability of the experimental procedure. None expressed concerns or disbelief regarding the role of both co-participants.

### Recordings

Recording sessions were performed individually in a quiet room. The experimenter familiarized the participants with the equipment and remained present in the room during the entire recording session. During each recording session, the participants' voice was recorded while reading three different types of sentences: *neutral* (“Danny went to work with his dad” and “Chad helped us on the beach”), *request* (“Please open the window”) and *command* (“Open the window immediately”). Participants were asked to read each sentence twice in a way consistent with their meaning. The sentences' order was randomized across participants. Participants' speech signals were recorded using a Sennheiser PC20 headset microphone (High Wycombe, United Kingdom). The microphone was positioned approximately 5 cm from the corner of the participant's mouth and connected directly to a desktop computer. Speech samples were recorded using the GoldWave program (Version 5.12, GoldWave, Inc., 2005), with a sampling rate set at 48 kHz (16 bit), mono channel (see Rochman and Amir, 2013 for a brief introductory tutorial on basic procedures for recording speech/voice and acquiring relevant acoustic measures).

### Manipulation

Participants were told that they will play an internet game “Cyberball” (see Williams et al., 2000), and were asked to visualize the game in order to practice visual metallization skills. On the computer screen, participants were presented with their own picture, as well as two other “participants” pictures (one man and one woman). When receiving the ball from one of the other two players, participants were required to indicate to whom they would like to throw the ball, by clicking on the appropriate player picture. In all conditions, the game lasted 30 ball tosses (approximately 5 min).

As already mentioned, there were three experimental conditions: Exclusion, Acceptance, and Popularity. In the Exclusion condition, the participant received three tosses (10%) in the beginning of the game. The rest of the time the tosses were interchanged between the two other presumed players while the participant was being ignored. In the Acceptance condition, the ball was passed equally frequently to all participants, resulting in the participant receiving 10 tosses (33%). In the Popularity condition the participant received 15 tosses (50%).

### Self-report measures

#### Basic needs threat questionnaire

(Zadro et al., 2006), contains 12 items assessing the effect of the game: *belonging* (e.g., “I felt like an outsider during the Cyberball game”), *control* (e.g., “I felt that I was able to throw the ball as often as I wanted during the game”), *self-esteem* (e.g., “I felt somewhat inadequate during the Cyberball game”), and *meaningful existence* (e.g., “I felt nonexistent during the Cyberball game”). All items are rated on a 5-point scale.

Consistent with previous research, the internal consistency of the need scale as a whole was very high (α = 0.93) (see Williams et al., 2000; Zadro et al., 2006). Additionally, the sub-scales of belongingness, control, self-esteem, and meaningful existence also exhibited adequate-to-high internal consistencies (alphas were 0.65; 0.85; 0.85; 0.77 respectively).

The questionnaire also contained two additional items regarding the “task” (e.g., “What percent of the throws were thrown to you?”, “To what extent were you included by the other participants during the game?”), and two 9-point bipolar scales assessing current mood (“negative/positive”) and feelings of rejection during the game (“accepted/rejected”).

#### Liebowitz sa scale-self-report

(LSAS-SR; Fresco et al., 2001), a 24-item self-report questionnaire measuring anxiety and avoidance in social or performance situations on a 0–3 scale. The LSAS-SR has been shown to have high internal consistency, strong convergent and discriminate validity, and high test-retest reliability (Baker et al., 2002; Fresco et al., 2001). In the present study, a Cronbach's α of 0.93 was obtained for the anxiety subscale and 0.90 for the avoidance subscale.

#### Beck depression inventory

(BDI; Beck et al., 1996), a 21-item, multiple-choice, self-report questionnaire that assesses affective, cognitive, motivational and somatic symptoms of depression. In the present study we obtained a Cronbach's α of 0.81 for this measure.

### Acoustic measures

*Mean Fundamental Frequency (mF0)* represents the rate of vibration of the vocal folds during phonation and speech. It is measured in Hz, and it is subjectively perceived as pitch. Men and women differ widely in mF0s, which is estimated to average around 220 Hz for women and 130 Hz for men in general (Peterson and Barney, 1952), as well as among Hebrew speakers (Most et al., 2000).

*Vocal intensity* reflects the amount of acoustic/vocal energy produced by the speaker and could be related to the effort used by the speaker to produce speech (Laukka et al., 2008). It is measured in decibels (dB), and it is subjectively perceived as loudness.

## Results

### Participants' characteristics

Table 1 presents means and standard deviations (in parentheses) of participants' characteristics. Participants (*n* = 104, 58 women) ranged in age from 17 to 35, with a mean age of 23.41 years (*SD* = 3.13). Participants' level of education ranged from 12 to 18 years, with a mean of 13.17 (*SD* = 1.54). Participants LSAS scores ranged from 0 to 123 with a mean score of 39.44 (*SD* = 21.28), and BDI scores ranged from 0 to 28 with a mean score of 6.54 (*SD* = 5.47). Participants were divided to high vs. low SA groups (HSA and LSA respectively) based on median split of LSAS at the time of the experiment. The mean LSAS score in the LSA group was 24 (*SD* = 10.39) and the mean LSAS score in the HSA group was 57.46 (*SD* = 15.83).

**Table 1:** **Means and standard deviation (in parentheses) of participants' characteristics in the exclusion, acceptance, and popularity conditions according to social anxiety (SA) group**.

	**Exclusion**		**Acceptance**		**Popularity**	
	**High SA N = 16**	**Low SA N = 19**	**High SA N = 17**	**Low SA N = 16**	**High SA N = 15**	**Low SA N = 21**
Age	24.37_a_	25.08_a_	23.00_a_	23.13_a_	22.60_a_	22.31_a_
	(4.30)	(3.89)	(2.03)	(2.33)	(1.8)	(2.79)
LSAS	63.50_a_	21.26_b_	52.18_a_	25.31_b_	57.00_a_	25.48_b_
	(19.55)	(10.62)	(8.29)	(10.55)	(16.66)	(10.06)
BFNE	18.06_a_	10.53_b_	14.94_a_	12.13_a_	19.33_a_	10.19_b_
	(9.18)	(6.68)	(7.37)	(6.6)	(7.04)	(5.72)
BDI	11.13_a_	4.00_b_	6.88_a_	5.81_a_	9.20_a_	3.71_b_
	(6.18)	(3.28)	(4.08)	(4.45)	(7.23)	(3.73)
% Throws	6.69_a_	6.89_a_	29.53_a_	28.41_a_	53.67_a_	50.29_a_
	(4.08)	(3.13)	(5.39)	(4.84)	(13.42)	(11.23)
Exclusion	4.19_a_	3.84_a_	1.24_a_	1.38_a_	1.070_a_	1.05_a_
	(0.65)	(0.96)	(0.44)	(0.62)	(0.26)	(0.22)
Ignore	4.31_a_	4.00_a_	1.35_a_	1.19_a_	1.07_a_	1.00_a_
	(0.60)	(0.94)	(0.49)	(0.40)	(0.26)	(0.00)
Mood	5.06_a_	6.53_b_	7.24_a_	7.63_a_	7.07_a_	7.67_a_
	(1.84)	(1.61)	(1.35)	(1.09)	(0.80)	(1.35)
Belonging	1.65_a_	1.88_a_	3.12_a_	3.33_a_	3.98_a_	3.78_a_
	(0.67)	(0.59)	(0.64)	(0.74)	(0.55)	(0.82)
Control	1.83_a_	2.39_b_	3.84_a_	4.02_a_	4.29_a_	4.21_a_
	(0.68)	(0.78)	(0.68)	(0.41)	(0.45)	(0.64)
Self-esteem	2.21_a_	3.30_b_	4.25_a_	4.39_a_	3.88_a_	4.57_b_
	(0.88)	(0.74)	(0.58)	(0.53)	(0.58)	(0.38)
Meaningful existence	1.97_a_	2.54_b_	4.14	4.27_a_	3.96_a_	4.35_b_
	(0.54)	(0.71)	(0.69)	(0.42)	(0.77)	(0.40)
Fundamental needs	1.92_a_	2.53_b_	3.84_a_	4.00_a_	4.02_a_	4.23_a_
	(0.54)	(0.52)	(0.41)	(0.36)	(00.48)	(0.31)
% Women	62.5_a_	31.60_a_	70.60_a_	62.50_a_	60.00_a_	52.40_a_

*Different subscripts (i.e., a, b) within each pair represent differences at 0.05 level, and identical subscripts(a, a) represent a lack of statistically significant difference*.

### Manipulation checks

In order to assess whether participants correctly perceived the number of throws they received, we conducted a Three-Way ANOVA with 3 (Condition: Exclusion, Acceptance, Popularity) × 2 (Group: HSA, LSA) × 2 (Gender: Men, Women). The analysis revealed the expected main effect of Condition, [*F*_(2, 91)_ = 250.32, *p* < 0.001, η^2^ = 0.85]. No other main effects or interactions were found (all *ps* > 0.36). Thus, it is concluded that participants correctly perceived whether they were excluded, accepted, or made popular in the game. Moreover, SA group did not affect the correct estimation of perceived tosses [*F*_(1, 91)_ = 0.62, *p* = 0.43].

In order to assess whether participants correctly labeled their experiences, we conducted a Three-Way MANOVA on exclusion, ignoring, and acceptance ratings, with 3 (Condition: Exclusion, Acceptance, Popularity) × 2 (Group: HSA, LSA) × 2 (Gender: Men, Women) as between-subject variables. The analysis revealed the expected main effect of Condition, [Wilks' Lambda *F*_(6, 178)_ = 59.53, *p* < 0.001, η^2^ = 0.67]. No other main effects or interactions were found (all *ps* > 0.93).

### Subjective self-report

**Figure 1:**
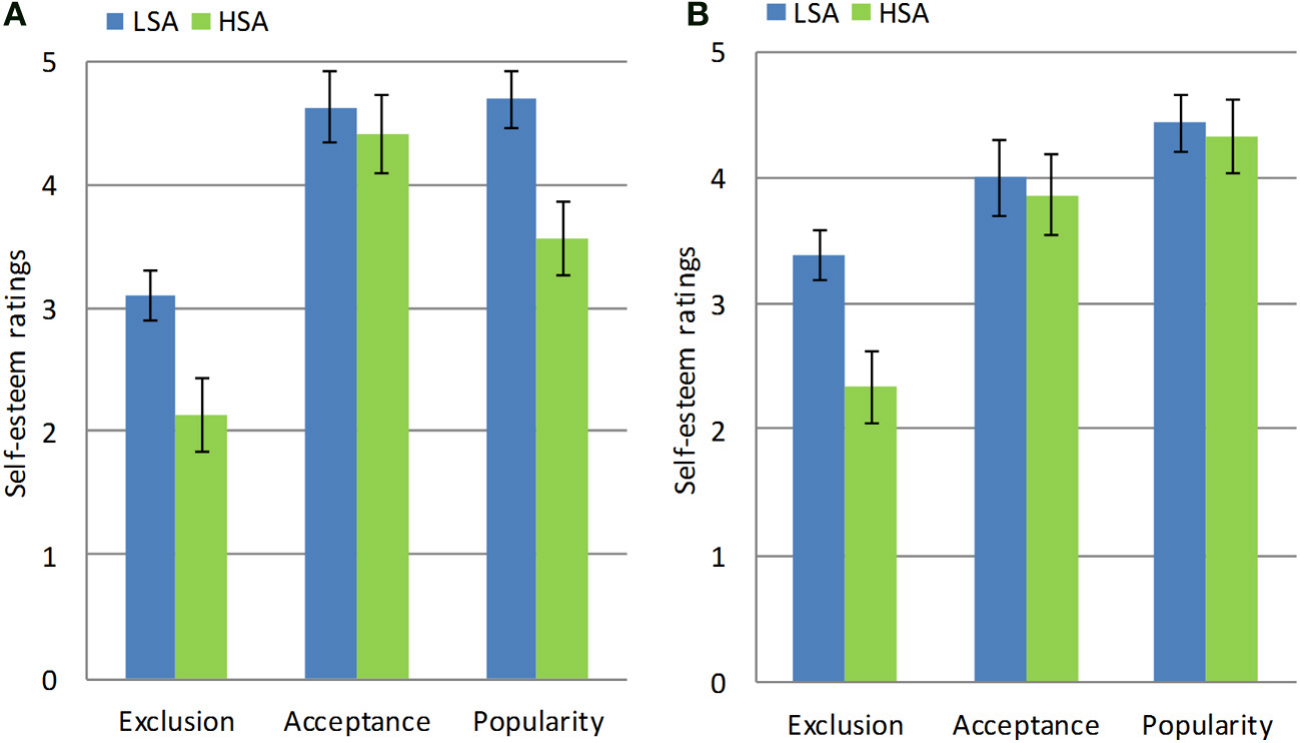
**Self-esteem measures of women (A) and men (B) in the high and low social anxiety groups following exclusion, acceptance, and popularity manipulations**. Error bars represent standard errors of the mean.

In the examination of the exclusion reactivity hypothesis and the impaired positivity hypothesis we included BDI as a covariate, as it was significantly related to measures of interest (*r* > −0.19, *p* = 0.05). Participant's subjective self-report measures according to Condition, SA group and Gender are presented in Figure 1.

#### The enhanced exclusion reactivity hypothesis

To test this hypothesis we first conducted an ANCOVA on mood ratings with 2 (Condition: Exclusion, Acceptance) × 2 (Group: HSA, LSA) × 2 (Gender: Men, Women) as between-subject variables, and BDI as a covariate. A main effect of Condition was found, such that participants in the Exclusion condition reported lower mood as compared to the participants in the Acceptance condition [*F*_(1, 59)_ = 14.78, *p* < 0.001, η^2^ = 0.20]. A main effect of Group was found, such that individuals in the HSA group reported lower mood than did individuals in the LSA group [*F*_(1, 59)_ = 5.69, *p* < 0.02, η^2^ = 0.08]. The effect for Gender approached significance, such that women reported lower mood than did men [*F*_(1, 59)_ = 3.39, *p* = 0.07, η^2^ = 0.05]. Inconsistent with our hypothesis, no Group × Condition interaction was found [*F*_(1, 59)_ = 1.18, *p* = 0.28]. No other main effects or interactions approached significance (all *ps* > 0.28).

Next, we conducted a MANCOVA on fundamental needs scales (i.e., belongingness, control, self-esteem, and life meaning), with 2 (Condition: Exclusion, Acceptance) × 2 (Group: HSA, LSA) × 2 (Gender: Men, Women) as between-subject variables, and BDI as a covariate. A main effect of Condition was found, such that participants in the Exclusion condition reported having lower needs scores (i.e., more need-threat) than did participants in the Acceptance condition [*F*_(4, 56)_ = 47.84, *p* < 0.001, η^2^ = 0.77]. Moreover, the effect of Group approached significance, [*F*_(4, 56)_ = 2.32, *p* = 0.068, η^2^ = 0.14], such that individuals with HSA tended to have lower needs scores than individuals with LSA. Finally, a Condition × Gender interaction was found, such that the difference in needs scores following Exclusion vs. Acceptance in women was greater than this difference among men [*F*_(4, 56)_ = 2.88, *p* < 0.03, η^2^ = 0.17]. In addition, consistent with our prediction, we found that, as compared to individuals with LSA, individuals with HSA reported lower self-esteem scores following Exclusion, as compared to the Acceptance conditions [*F*_(1, 59)_ = 4.84, *p* < 0.03, η^2^ = 0.09]. No other main effects or interactions approached significance (all *ps* > 0.27).

#### The impaired positivity hypothesis

In order to examine this hypothesis, we first conducted an ANCOVA on mood ratings, with 2 (Condition: Popularity, Acceptance) × 2 (Group: HSA, LSA) × 2 (Gender: Men, Women) as between-subject variables, and BDI as a covariate. No main effect of Condition was found [*F*_(1, 60)_ = 0.3, *p* = 0.59]. A main effect of BDI was found, such that higher depression was associated with lower mood [*F*_(1, 60)_ = 4.02, *p* < 0.05, η^2^ = 0.06]. A Condition × Gender interaction approached significance [*F*_(1, 60)_ = 3.86, *p* = 0.054, η^2^ = 0.06]. Importantly, this Two-Way interaction was modified by a three way interaction between Condition, Gender and Group [*F*_(1, 60)_ = 5.69, *p* < 0.02, η^2^ = 0.08]. We examined the differences for men and women separately. Men with HSA tended to report *higher* mood ratings following Popularity compared to Acceptance. At the same time, men with LSA did not evidence any difference in mood between the conditions [*F*_(1, 22)_ = 2.36, *p* = 0.14]. In contrast, women with HSA tended to report *lower* mood ratings following Popularity as compared to Acceptance while LSA women did not evidence any difference in mood between the conditions [*F*_(1, 37)_ = 2.85, *p* = 0.1]. In other words, the impaired positivity hypothesis was supported for women only, while men with HSA appeared to exhibit enhanced affective reactivity to Popularity. No other main effects or interactions were significant (all *ps* > 0.23).

Next, we conducted a MANCOVA on fundamental needs scales, with 2 (Condition: Popularity, Acceptance) × 2 (Group: HSA, LSA) × 2 (Gender: Men, Women) as between-subject variables, and BDI as a covariate. A main effect of Condition was found, such that participants in the Popularity condition reported having higher needs scores (i.e., less need-threat) than did participants in the Acceptance condition [*F*_(4, 57)_ = 4.57, *p* < 0.003, η^2^ = 0.24]. Moreover, a significant effect of Group was found, such that individuals with HSA tended to have lower needs scores than individuals with LSA [*F*_(4, 57)_ = 2.66, *p* < 0.04, η^2^ = 0.16]. Finally, a Condition × Gender interaction was found, such that the differences in needs scores for women in the Popularity vs. Acceptance condition were smaller than they were for men [*F*_(4, 57)_ = 3.38, *p* < 0.01, η^2^ = 0.19].

An examination of the effects of the Self-esteem needs did not identify the predicted Group × Condition interaction [*F*_(1, 60)_ = 2.78, *p* = 0.1]. However, a Three-Way Group × Condition × Gender interaction was found [*F*_(1, 60)_ = 4.54, *p* < 0.04, η^2^ = 0.07]. We examined the differences for men and women separately. Men with both high and low SA level tended to report higher self-esteem ratings following Popularity as compared to Acceptance [*F*_(4, 19)_ = 2.59, *p* = 0.07]. Women in the HSA group reported lower self-esteem rating following Popularity as compared to Acceptance, while women in the LSA group did not evidence any difference in self-esteem between the conditions [*F*_(4, 34)_ = 5.53, *p* < 0.002, η^2^ = 0.41]. Again, the impaired positivity hypothesis was supported for women, but not for men. No other main effects or interactions were significant (all *ps* > 0.23).

### Acoustic measures

Acoustic analyses were performed using Praat© software (Version 4.1.2, Boersma and Weenink, 2009). Two parameters were extracted (a) mF0: mean fundamental frequency; and (b) Vocal intensity: mean speech vocal intensity. Only command and neutral utterances were analyzed, as we did not generate specific predictions for the request utterances. In light of our hypotheses, we focused on the main effect and interactions involving Group.

For each acoustic parameter, outliers of more than three standard deviations above or below the mean were excluded from the analysis (as in Weeks et al., 2011). Means and standard deviation for each parameter in each Sentence-type and Gender are presented in Table 2. Because there was no correlation between BDI and mF0 or vocal intensity, BDI was not included in the analyses.

**Table 2:** **Means and standard deviation (in parentheses) of acoustic parameters recorded after exclusion and popularity conditions according to social anxiety (SA) group and gender**.

	**Low SA**			**High SA**		
	**T1**	**T2**	**Change**	**T1**	**T2**	**Change**
**EXCLUSION CONDITION**						
Neutral sentences						
mF0 (M)	123.23 (5.42)	122.12 (5.3)	−0.53 (2.01)	143.86 (7.98)	146.77 (7.79)	1.83 (2.99)
mF0 (W)	192.33 (7.98)	191.06 (7.79)	−1.27 (2.73)	208.69 (6.18)	209.89 (6.04)	0.08 (2.23)
Vocal intensity (M)	72.34 (1.28)	70.89 (1.14)	−1.87 (1.68)	69.16 (1.89)	69.55 (1.69)	0.65 (2.48)
Vocal intensity (W)	70.87 (1.89)	68.73 (1.69)	−2.14 (2.27)	70.03 (1.46)	66.47 (1.31)	−3.33 (1.85)
Command sentences						
mF0 (M)	150.4 (6.78)	140.25 (6.77)	−9.57 (3.37)	162.42 (9.99)	168.89 (9.96)	7.83 (5.00)
mF0 (W)	228.76 (9.99)	214.5 (9.96)	−14.26 (4.56)	234.61 (7.74)	235.12 (7.72)	−1.22 (3.73)
Vocal intensity (M)	76.89 (1.42)	72.92 (1.22)	4.03 (1.27)	71.1 (2.09)	75.74 (1.79)	−4.89 (1.89)
Vocal intensity (W)	75 (2.09)	75.02 (1.79)	−0.02 (1.72)	72.76 (1.62)	71.15 (1.39)	1.53 (1.41)
Request sentences						
mF0 (M)	130.32 (5.98)	130.9 (5.92)	0.73 (3.19)	149.85 (8.8)	157.62 (8.71)	11.48 (4.74)
mF0 (W)	207.89 (8.8)	204.15 (8.71)	−3.74 (4.32)	220.6 (6.82)	212.06 (6.75)	−7.44 (3.53)
Vocal intensity (M)	70.24 (1.38)	69.38 (1.2)	−1.34 (1.50)	66.4 (2.036)	72.34 (1.769)	7.38 (2.23)
Vocal intensity (W)	70.15 (2.04)	71.06 (1.77)	0.91 (2.04)	67.37 (1.58)	66.51 (1.47)	−0.97 (1.66)
**POPULARITY CONDITION**						
Neutral sentences						
mF0 (M)	114.18 (6.18)	115.74 (6.04)	1.16 (2.36)	121.8 (7.979)	124.38 (7.79)	1.32 (2.99)
mF0 (W)	196.58 (5.89)	198.16 (5.76)	1.58 (2.01)	194.24 (6.91)	198.78 (6.75)	4.54 (2.36)
Vocal intensity (M)	69.32 (1.46)	71.1 (1.31)	−0.23 (1.96)	70.52 (1.89)	69.5 (1.69)	3.02 (2.448)
Vocal intensity (W)	68.76 (1.4)	69.19 (1.24)	0.43 (1.68)	69.72 (1.54)	69.68 (1.38)	0.41 (1.96)
Command sentences						
mF0 (M)	129.09 (7.74)	128.98 (7.72)	−1.10 (3.95)	143.91 (9.99)	147.63 (9.96)	3.16 (5.00)
mF0 (W)	215.09 (7.38)	212.28 (7.36)	−2.80 (3.37)	229.91 (8.65)	226.22 (8.63)	−3.69 (3.95)
Vocal intensity (M)	72.37 (1.62)	71.87 (1.39)	0.44 (1.49)	74.91 (2.09)	76.26 (1.79)	1.24 (1.89)
Vocal intensity (W)	72.72 (1.55)	71.1 (1.32)	1.62 (1.27)	74.35 (1.71)	71.95 (1.46)	2.34 (1.49)
Request sentences						
mF0 (M)	125.82 (6.82)	119.86 (6.75)	−2.74 (3.74)	128.93 (8.8)	134.22 (8.71)	6.12 (4.74)
mF0 (W)	202.36 (6.5)	202.89 (6.43)	0.54 (3.19)	207.24 (7.62)	210.59 (7.54)	3.34 (3.74)
Vocal intensity (M)	68.01 (1.58)	67.18 (1.37)	−2.34 (1.76)	68.12 (2.04)	69.31 (1.77)	1.27 (2.23)
Vocal intensity (W)	68.43 (1.5)	67.76 (1.31)	−0.67 (1.50)	69.08 (1.66)	68.59 (1.44)	−0.26 (1.76)

#### Vocal insecurity following exclusion hypothesis

**Figure 2:**
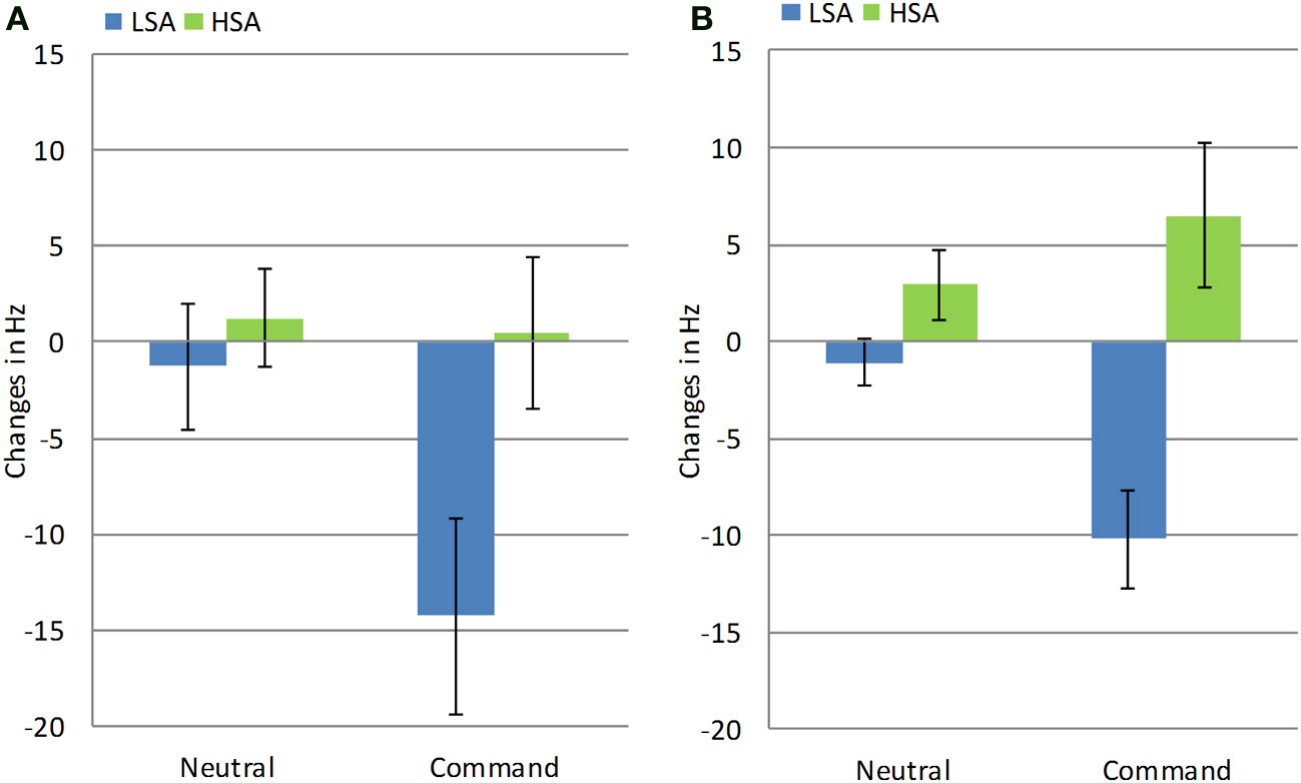
**Changes in mF0 following exclusion in high and low socially anxious women (A) and men (B)**. Error bars represent standard errors of the mean.

**Figure 3:**
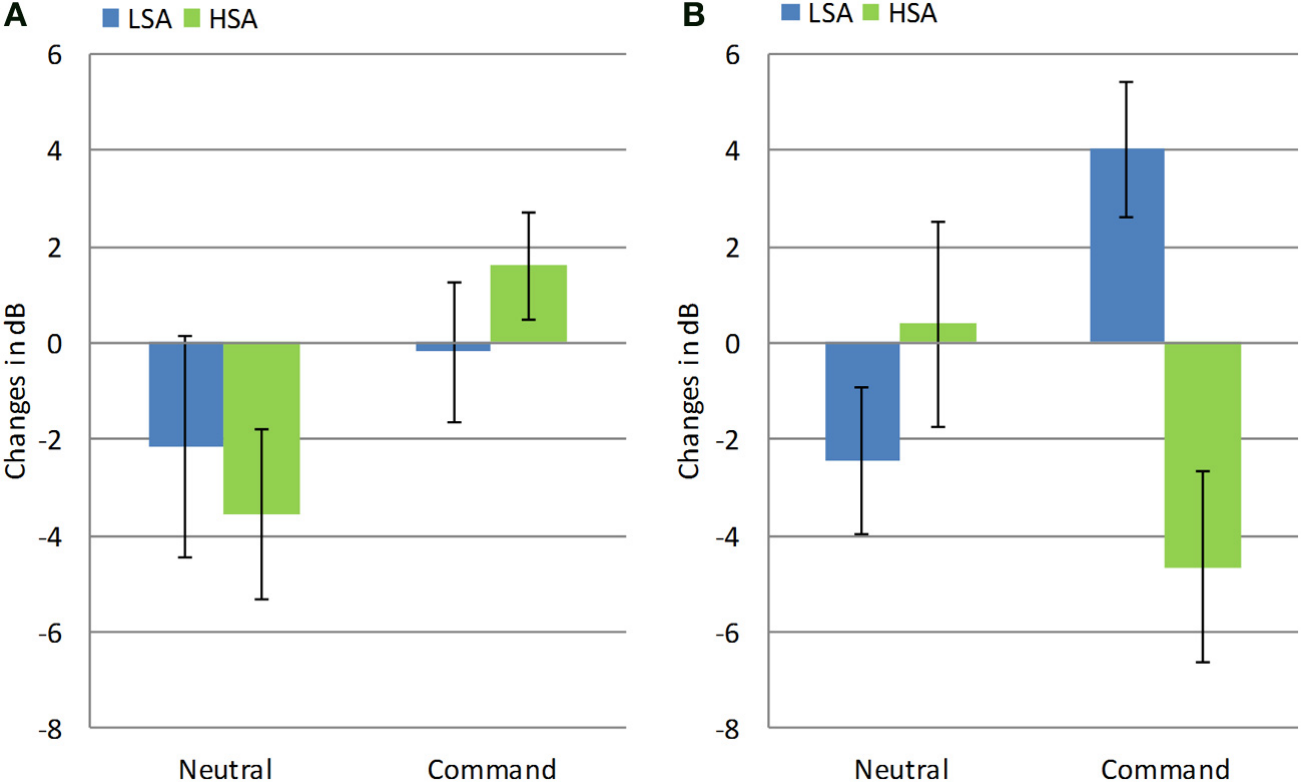
**Changes in vocal intensity following exclusion in high and low socially anxious women (A) and men (B)**. Error bars represent standard errors of the mean.

In order to test this hypothesis, we conducted two separate repeated measures analyses on mF0 and vocal intensity. To this end, a difference score between the pre- and post-manipulation measurement was computed for each participant in the Exclusion condition for mF0 and vocal intensity of command and neutral utterances. Changes in acoustic parameters following Exclusion according to SA group and Gender are presented in Figures 2, 3.

First, an ANOVA on mF0 was conducted with Gender (Men, Women) and Group (LSA, HSA) as between-subject variables, and Sentence-type (Neutral, Command) as a within-subject variable. A significant main effect of Group was found, such that overall, individuals with LSA exhibited a decrease in mF0 compared to individuals with HSA, for whom mF0 increased [*F*_(1, 31)_ = 13.26, *p* < 0.001, η^2^ = 0.30]. Importantly, and consistent with our hypothesis, this main effect was modified by a significant Sentence-type × Group interaction, such that only individuals with LSA lowered their mF0 from neutral to command sentences [*F*_(1, 31)_ = 17.33, *p* < 0.001, η^2^ = 0.36]. Because of the significant differences in mF0 between men and women, we examined these findings separately for each gender. Results confirmed that this interaction was significant for both men [*F*_(1, 17)_ = 13.23, *p* < 0.002, η^2^ = 0.44] and women [*F*_(1, 14)_ = 5.95, *p* < 0.03, η^2^ = 0.30].

Then, a similar ANOVA was conducted for the vocal intensity measure. A significant Three-Way Sentence-type × Gender × Group interaction was found [*F*_(1, 30)_ = 6.93, *p* < 0.001, η^2^ = 0.19]. Consistent with our hypothesis, LSA men increased their vocal intensity, while HSA men decreased their vocal intensity in command utterances, as compared to neutral sentences [*F*_(1, 16)_ = 7.26, *p* < 0.02, η^2^ = 0.31]. In contrast, both HSA and LSA women exhibited a greater increase in vocal intensity for command sentences as compared to neutral sentences [*F*_(1, 14)_ = 4.6, *p* < 0.05, η^2^ = 0.25].

#### Vocal confidence following popularity hypothesis

In order to test this hypothesis, we conducted two separate repeated measures analyses on mF0 and vocal intensity. Similarly to the Exclusion condition, we used the difference score between the pre- and post-Popularity measures in acoustic parameters (mF0, vocal intensity) for command and neutral utterances.

An ANOVA on mF0 was conducted with Gender (Men, Women) and Group (LSA, HSA) as between-subject variables, and Sentence-type (Neutral, Command) as a within-subject variable. No significant effects or interactions were identified (all *ps* > 0.13). A similar ANOVA was conducted on the vocal intensity measures, with no significant main effects or interactions (all *ps* > 0.25).

## Discussion

The present study examined reactivity to changes in belongingness based on subjective and expressive (implicit) measures in individuals high and low on a self-report measure of SA. First, our exclusion-reactivity hypothesis was partially supported. Our results support previous findings that threat to belongingness has a general negative effect on individuals, but that, on most measures, the immediate effect of exclusion is not associated with individual differences (Zadro et al., 2006; Oaten et al., 2008; Williams, 2009; but see also Wesselmann et al., 2012a,b). Specifically, we did not find that individuals with HSA reported lower mood or higher threat of their fundamental needs following exclusion (as compared to acceptance), as compared to individuals with LSA. However, consistent with our hypothesis, we found that, as compared to individuals with LSA, individuals with HSA were more affected by exclusion (than acceptance) condition on measures assessing self-esteem. Importantly, these findings held while controlling for significant effects of depressive symptoms severity. Thus, while there were no differences in the way that HSA and LSA individuals perceived the reality of the interaction (i.e., both groups estimated number of throws equally accurately), HSA individuals reported lower self-esteem following exclusion than following acceptance than did LSA individuals. These results are consistent with previous findings demonstrating that SA in children was associated with greater changes in self-esteem following rejection (Reijntjes et al., 2011). Thus, the present findings are broadly consistent with Leary's view of SA as possessing an over-sensitive sociometers (e.g., Leary and Jongman-Sereno, in press).

Second, we tested the impaired positivity hypothesis, which postulated that individuals with HSA will report attenuated subjective reactions to popularity compared to acceptance. Our impaired positivity hypothesis was supported for women, but not for men. Specifically, we found that while SA did not affect men's self-esteem ratings in response to popularity as opposed to acceptance, HSA, but not LSA women, reported decreases in mood and in self-esteem. Importantly, men with HSA were found to be more affectively responsive to popularity than to acceptance as opposed to men with LSA. It appears that HSA men are more dependent on external feedback than are LSA men. These findings support and extend the research showing that gender exerts a significant effect on interpersonal relationships (e.g., Benenson, 1990; Kwang et al., 2013). Specifically, it is possible that while social visibility (being at the center) does not carry negative costs for men, such visibility may incur negative consequences for women (e.g., Cillessen and Borch, 2006). Alternatively, it is also possible that popularity in the ball-tossing game carries different (and more positive) connotations for men than for women.

Third, we postulated that individuals with HSA would exhibit a pattern of vocal insecurity following exclusion, whereas individuals with LSA would not exhibit this pattern. This hypothesis was mostly supported by our findings. Specifically, we found that HSA men exhibited an increase in mF0 and a decrease in vocal intensity in command sentences. In contrast, LSA men exhibited an opposite pattern: they evidenced a decrease in mF0 and an increase in vocal intensity. Similarly, HSA women uttered command sentences in higher mF0 than did LSA women. Taken together, these findings suggest that after experiencing exclusion, men, and to a somewhat lesser extent, women, with LSA exhibit a confident and dominant pattern of responses, while individuals with HSA exhibit an insecure pattern.

Fourth, we tested the impaired confidence hypotheses, according to which HSA individuals are expected to exhibit a less pronounced increase in vocal confidence than those with LSA after experiencing a popularity condition. This hypothesis was not supported by our data.

### Social exclusion: reparative repertoire

When interpersonal status-quo is threatened, due to social exclusion or rejection, the need to take reparative action arises. Such a need is likely to mobilize various subsystems, energize behavior, attune the sensitivity of the cognitive system to signals of acceptance or rejection, and influence motivation and behavior. Previous studies have documented that social exclusion may lead to distinct types of responses. These include social coldness/avoidance (e.g., DeWall and Baumeister, 2006; Twenge et al., 2007), affiliation (Maner et al., 2007; Dewall et al., 2009) and aggression (Twenge et al., 2001; DeWall et al., 2010). Insofar as acoustic parameters are seen as proxy for interpersonal strategies, our study suggests that, some individuals react to social exclusion by adopting strategies aimed for restoring social status, while others may react by “profile lowering” and utilization of behaviors typically associated with submissiveness and deference.

The interpersonal circumplex (e.g., Wiggins, 1979) conceptualizes the realm of social behaviors as consisting of two axes: dominance (i.e., power, competence, agency) and affiliation (i.e., warmth, love, communion). When examined through this prism, social exclusion can either heighten or lower the desire to affiliate, and the motivation to restore social rank. This conceptualization brings the rather disparate literature of reactions to exclusion under a unified theoretical umbrella, suggesting that exclusion (and possibly popularity) may lead to the use of strategies for increasing social rank, and not only those intended to regain social acceptance. In addition, social exclusion may lead to the simultaneous employment of several types of coping strategies, as people may increase their social visibility while also increasing the affiliative efforts on the one hand, or signal deference and social withdrawal on the other hand (see also Powers and Heatherton, 2012). Considered in concert, these findings are suggestive of the great flexibility and diversity of responses to social exclusion.

### Exclusion and social anxiety

In this study we found that vocal characteristics of command and neutral sentences provided cues for changes in belongingness status, and that individual differences (gender, SA) modulated these effects. Specifically, we suggest that HSA individuals respond to social exclusion by using submissive tactics. These findings are in line with previous studies, which similarly found that individuals with HSA report using more submissive behaviors and endorse more submissive cognitions than individuals with LSA (Aderka et al., 2009; Weeks et al., 2011). In addition, other studies have found that individuals high in SA were rated as less dominant, and that HSA women made greater efforts to minimize interpersonal disharmony than did LSA women, by using more appeasement statements (Oakman et al., 2003).

These findings lent further support to theoretical accounts which place concerns with social rank and power at the core of SA (e.g., Gilbert, 2001; Gilbert and Trower, 2001; Mineka and Öhman, 2002; Johnson et al., 2012; Gilboa-Schechtman and Shachar-Lavie, 2013). HSA individuals opt for submissive or deferring responses when faced with social threats—either exclusion or defeat. Future studies may explore whether, and under what conditions, social exclusion/rejection in HSA individuals leads to deficits in affiliative behavior, deficits in assertive behaviors, or general social withdrawal.

Recent studies focused on the neural correlates of interpersonal exclusion in individuals with psychopathology (e.g., Maurage et al., 2012). Specifically, Maurage and colleagues found that, as compared to controls, individuals with alcohol dependence, exhibited increased activation in brain “reactivity” areas (i.e., areas usually associated with social exclusion feelings such as dorsal anterior cingulate cortex, insula) as well as decreased activation in areas associated with regulations of those feelings (e.g., middle frontal gyrus and inferior frontal gyrus). Extending these studies to examine the neural correlates of social exclusion (and possibly social rank loss) in SA may strengthen our understanding of core mechanism(s) of this disorder.

The present findings extend existing research in several ways. First, while previous research focused mostly on affiliative responses following exclusion (e.g., Maner et al., 2007; Mallott et al., 2009; Buckner et al., 2010; Tai et al., 2011), we focused on responses connoting dominance and submissiveness. Second, we examined expressive interpersonal responses. The emphasis on production, rather than perception of social signals, is essential for evaluating the impact of behaviors of socially anxious individuals on their chances of creating a supportive and respectful interaction. In addition to conveying the speaker's emotional states, vocal expressions may also serve as a signal to the listener, serving as an appeal for reaction (Laukka and Elfenbein, 2011). Such expressions modulate and coordinate interpersonal interactions. Third, we found that the pattern of affective, cognitive, and behavioral response was specific to SA, rather than emerging from concomitant depressive symptoms. This emphasizes the impaired reactions to exclusion as a core feature of SA.

### Popularity, social anxiety, and gender

Evolutionary and interpersonal perspectives converge in suggesting that social stress arises in response to changes and modulation in social standing and social fortunes (e.g., Gilbert and Trower, 2001; Alden and Taylor, 2004). While research so far has focused on the examination of social threats (e.g., public speaking) and negative social events (e.g., exclusion, rejection), we examined the after-effects of exclusive social attention (popularity). Consistent with impaired positivity accounts, our findings suggest that the effects of enhanced social attention tend to be negative for women high (but not low) in SA. The mood and self-esteem of women with HSA decreased in situations of enhanced attention, compared to situations of equal attention (see also Gilboa-Schechtman et al., 2000; Gilbert and Trower, 2001; Alden et al., 2008; Weeks, 2010; Gilboa-Schechtman et al., 2013a,b). In contrast, men did not exhibit the predicted negatively biased reactivity to popularity. Instead, in that condition, men tended to exhibit an enhanced affective reactivity, supporting a high contingency of social esteem and external approval on SA (Reijntjes et al., 2011; Leary and Jongman-Sereno, in press). It is possible that, while no differences in subjective experience following exclusive social attention are reported by men high and low in SA, brain activation measures may unveil a different, more sensitive, pattern (for a similar argument, see Eisenberger and Lieberman, 2004).

### Limitations and future directions

Several limitations of our study should be noted. First, while the popularity condition affected the perceptions and the fundamental needs of our participants, it did not affect their mood ratings or the acoustic measures. We take these findings to mean that our popularity manipulation is a less powerful counterpart to the exclusion condition. Future research may attempt to enhance the effectiveness of popularity manipulation by using alternative procedures. Such alternatives could include the “survivor game” used by Reijntjes et al. (2011), the interpersonal rejection paradigm, as in Mallott et al. (2009), or a modification of the Cyberball procedure that would include additional participants, to enhance the difference between the acceptance and the popularity conditions. Second, we used only post-manipulation measures of mood, as typically performed in previous studies with Cyberball. Thus, we could only compare both exclusion and popularity conditions to the acceptance condition. Such comparisons are clearly less sensitive than within-subject comparisons. Future studies may use other manipulations allowing the assessment of pre- and post-mood measures. Third, our findings need to be replicated with spontaneous, rather than planned speech. Spontaneous speech is likely to involve increased task demands, as the speaker is concerned with the content of communication as well as with its manner. This may lead to greater or more pervasive disruption in vocal characteristics. Fourth, in this study we focused on a limited number of acoustic parameters. A more comprehensive examination of a wide range of expressive tactics (vocal, postural, facial) would enrich our understanding of the ways in which humans express intentions and emotions. Fifth, our sample size was rather small, likely restricting our ability to detect some individual differences. Sixth, our results need to be replicated in a clinical population. While there is considerable evidence that SA and SAD form a continuum (e.g., Ruscio, 2010; Haslam et al., 2012), it is possible that individuals with clinical levels of SA exhibit qualitatively different forms of impairment. Moreover, future studies could profit from a differentiation between the effects of social and generalized anxiety on responses to changes in belongingness. Finally, in our study we examined the effects of threats to belongingness. An extension of the present finding to other domains, such as threats to social status (e.g., winning or losing a competition), would allow a greater understanding of the response to changes in interpersonal fortunes in SA.

### Summary and conclusions

Despite these limitations, we believe that our study makes several contributions. First, we show that a brief manipulation of exclusion exerts significant and differential effects on vocal expression, which can be quantified objectively. Indeed, our study is the first to suggest that social exclusion affects expressive interpersonal signals. Second, we argue that vocal changes exhibited by highly socially anxious individuals (especially males) are related to dominance expression impairment. Taken together with previous research on vocal properties of speech in socially anxious individuals (e.g., Weeks et al., 2012; Galili et al., 2013) our data suggest that vocal parameters of speech, especially mF0, may be used as objective markers of SA. Third, our data point to the hypersensitivity of social rank biobehavioral system functioning in SA (see also Johnson et al., 2012). In fact, reactivity to changes in social fortune may emerge as a core vulnerability in SA (see also Levinson et al., 2013). Indeed, such a conceptualization of SA may inform interventions which can be designed to decrease the reactivity and increase the adaptability of socially anxious individuals' response to changes in belongingness and in social rank. Fourth, significant differences in the subjective reactions of socially anxious men and women to changes in belongingness were found. These findings are consistent with evolutionary and interpersonal accounts of SA and highlight the importance of examining the effects of SA and gender on expressive and subjective reactions to events connoting social acceptance and ascendance. The examination of SA from the perspective of basic psychological systems may offer a new, theory-based approach to the nosology and treatment of this highly prevalent anxiety disorder.

## Author contributions

Eva Gilboa-Schechtman was responsible for the design of the study, supervised the running of the participants, performed the majority of data analyses, and wrote the study for publication. Lior Galili assisted in the running of the study, analysis of the vocal data and write-up. Yair Sahar assisted in data analyses and write-up. Ofer Amir supervised the vocal analysis and assisted in write-up.

### Conflict of interest statement

The authors declare that the research was conducted in the absence of any commercial or financial relationships that could be construed as a potential conflict of interest.
